# Auditory Subjective-Straight-Ahead Blurs during Significantly Slow Passive Body Rotation

**DOI:** 10.1177/20416695211070616

**Published:** 2022-01-05

**Authors:** Akio Honda, Sayaka Tsunokake, Yôiti Suzuki, Shuichi Sakamoto

**Affiliations:** Department of Information Design, Faculty of Informatics, 13064 Shizuoka Institute of Science and Technology, Fukuroi, Japan; Research Institute of Electrical Communication, Tohoku University, Sendai, Japan; Research Institute of Electrical Communication, Tohoku University, Sendai, Japan; Research Institute of Electrical Communication, Tohoku University, Sendai, Japan

**Keywords:** listener movement, sound localization, spatial hearing, multisensory integration

## Abstract

This paper reports on the deterioration in sound-localization accuracy during listeners’ head and body movements. We investigated the sound-localization accuracy during passive body rotations at speeds in the range of 0.625–5 °/s. Participants were asked to determine whether a 30-ms noise stimuli emerged relative to their subjective-straight-ahead reference. Results indicated that the sound-localization resolution degraded with passive rotation, irrespective of the rotation speed, even at speeds of 0.625 °/s.

Spatial hearing is considered as a multisensory-integration process involving self-motion (Suzuki et al., 2020). Earlier studies demonstrated that the listener's head/body movement facilitates sound localization (Honda et al., 2007, 2013, 2018; Iwaya et al., 2003; Kawaura et al., 1989/1991; Thurlow & Runge, 1967; Wallach, 1939), but recent reports have shown that sound localization accuracy deteriorates during the listener's head/body rotation (Cooper et al., 2008; Honda et al., 2016, 2020).

It is interesting that these deterioration effects are independent of rotation velocities in the range of 5–60 °/s (Honda et al., 2016, 2020). Recently, Honda et al. (2020) investigated the effect of passive body rotations on the accuracy of the subjective-straight-ahead (SSA) orientation of listeners. In this study, the participants were asked to keep their heads still while their chairs were rotated at speeds of 5, 10, and 20 °/s. The rotating-chair experiment revealed a significant reduction in the sound-localization accuracies measured irrespective of the rotational speed down to 5 °/s. These results indicate that the deterioration in sound localization is not due to bottom-up effects such as ear-input blurring.

If the deteriorating effect persists irrespective of the speed at lower speeds than previously tested, this phenomenon may be attributed to a top-down effect, such as the participant's conscious perception of motion. To test this hypothesis, we conducted an experiment with the aim of gaining insights regarding the SSA-relative sound-localization resolution, considering passive body rotation speeds below 5 °/s. This issue has not been discussed extensively in literature.

Eight normal-hearing participants (six males and two females; aged 21–23) were recruited for the experiments performed in a dark anechoic room (Figure 1).

**Figure 1.**
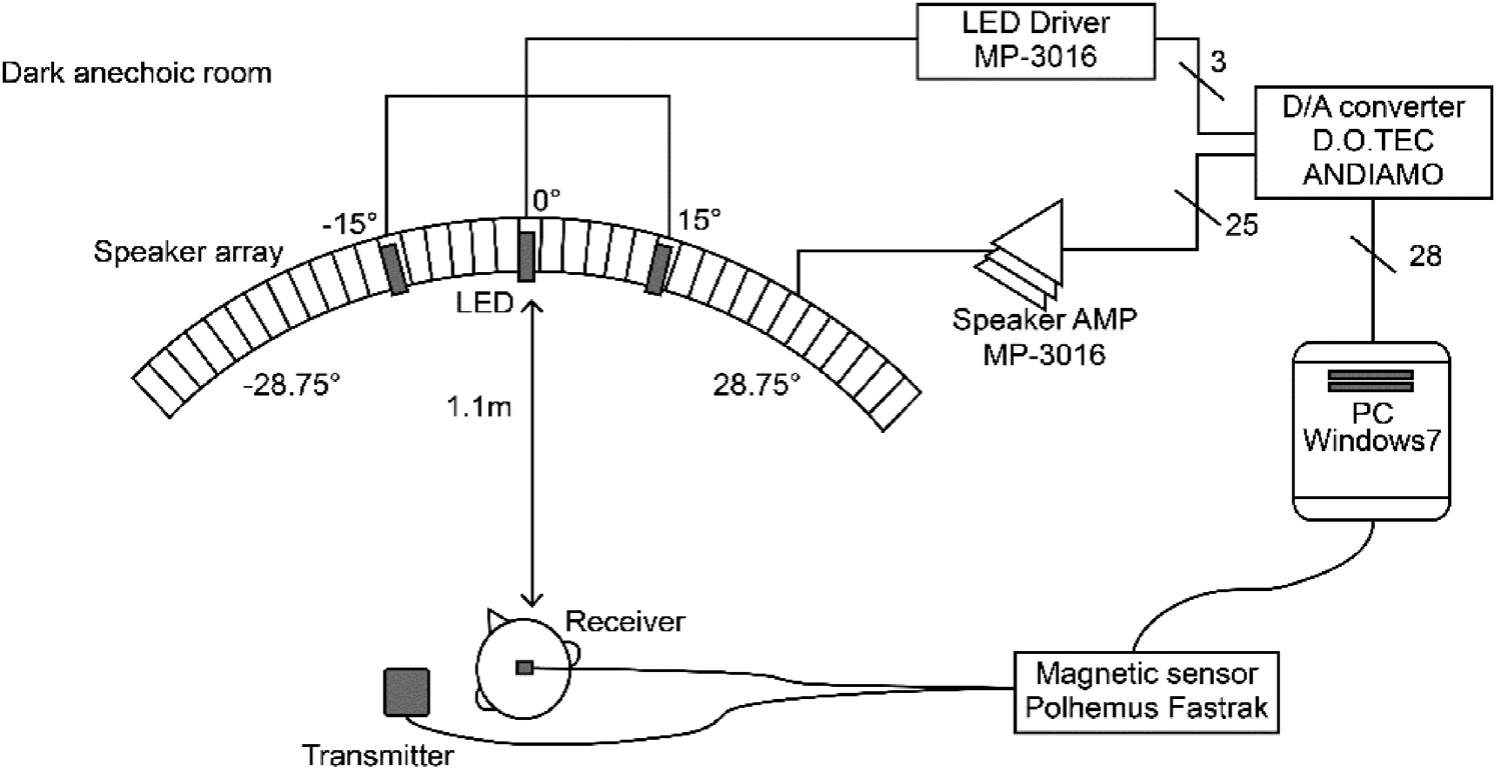
Experimental setup considered in this study. Sound bursts were generated using a circular-array (radius = 1.1 m; range = ±28.75°) of 30-mm loudspeakers (Hosiden 0254 7N101) separated by a 2.5° angular spacing. No special devices were used to restrict the participants’ head movements, and their head positions were monitored using a magnetic sensor placed at the top of their heads.

Two conditions—still- and rotating-chair—were considered during the experiments. Under both conditions, the participants were asked to judge the azimuthal direction of the sound image (left or right) relative to their SSA (i.e., the two-alternative forced choice (2AFC) approach). The acoustic stimulus comprised 1/3 octave-band noise with a 1-kHz center frequency. Each stimulus lasted 30 ms, including rise and decay times of 5 ms each. The A-weighted sound pressure level, when the band-noise was presented steadily, was set to 65 dB. The location of the acoustic stimulus was selected using the randomized maximum likelihood adaptation method (Takeshima et al., 2001). This study was approved by the ethics committee of the Research Institute of Electrical Communication at Tohoku University.

In the still-chair condition, the participants remained seated on the chair facing 0° and gazed at the LED, which was lit for 1 s, while keeping their heads still. Subsequently, the LED was switched off, and the acoustic stimulus was deployed from one loudspeaker located within ±11.25° of the participant's SSA. Next, the participants were asked to determine the direction from which the stimulus was deployed. One hundred such trials were performed.

In the rotating-chair condition, the participants remained seated on the chair facing ±15° and gazed at the LED for 1 s while keeping their heads still. After the LED was switched off, the chair was rotated at a speed of 0.625, 1.25, 2.5, or 5°/s to align with the 0° direction. After 4 s from the commencement of rotation, the acoustic stimulus was deployed from one loudspeaker located within ±15° of the participant's SSA. Once again, the participants were asked to determine the direction of origin of the stimulus. Ten similar sessions were performed for each participant, resulting in 640 trials (2 rotation directions × 4 rotation speeds × 80 trials). The rotation direction and speed were randomly selected.

The total number of left (0) and right (1) judgments under each condition were recorded. We then plotted the correct answering rate as a function of the angular distance between the loudspeaker position and the physical-straight-ahead of the observer. The point of subjective equality, i.e., the point of SSA (PSSA), is defined as the 50% point on the psychometric function for each condition for each participant. Moreover, the just noticeable difference (JND) of the PSSA for each condition is defined as the difference between the 50% point and the 75% point on the psychometric function. To estimate these points, we used the normal cumulative distribution function as the model psychometric function and numerically estimated its parameters (mean *m* and variance *σ*^2^). The function was fitted to the plots using maximum likelihood fitting (Ogura et al., 1989). Thus, the PSSA is the mean *m* of the estimated normal cumulative distribution function. Moreover, the JND of the PSSA is given by 0.6745*σ*, where *σ* denotes the estimated standard deviation, because the estimated psychometric function (the fitted normal cumulative distribution function) crosses 0.75 at the point *m* + 0.6745*σ*. One participant was excluded from the analysis owing to non-estimation of reasonable psychometric functions.

The one-way repeated-measure analysis of variance (ANOVA) was applied to the PSSA considering the rotational speed as a factor. As observed, the effect of the rotational speed was not significant (*F* (4, 24) = 1.41, n.s) (Figure 2). Interestingly, application of the said ANOVA to the JND demonstrated a significant rotational-speed effect (*F* (4, 24) = 9.26, *p *<* *.0001). Moreover, the post-hoc analysis (Ryan's method, *ps *<* *.05) yielded a small JND value under the still-chair condition compared to the rotating-chair scenario (Figure 3).

**Figure 2.**
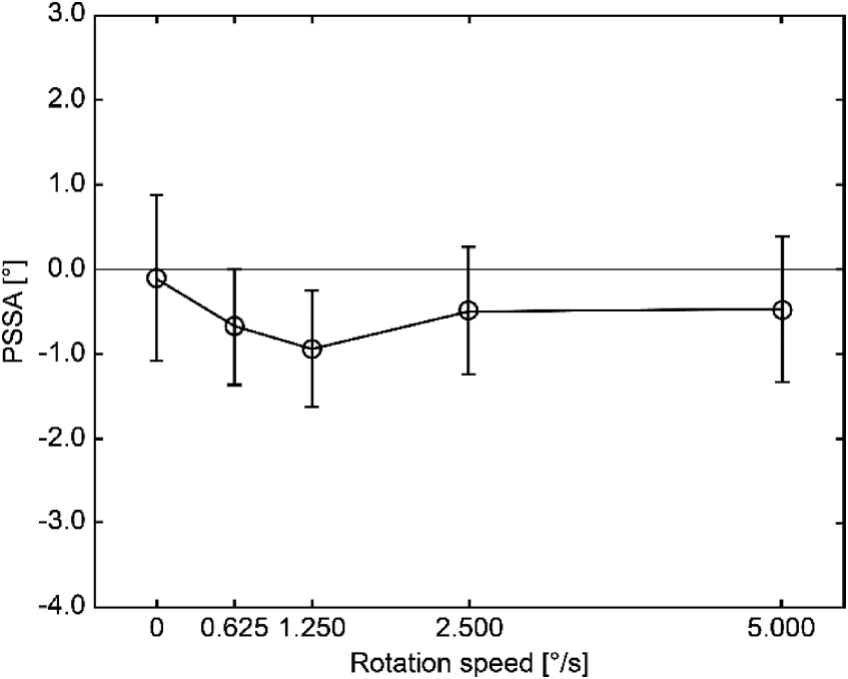
Average PSSA and standard error values.

**Figure 3.**
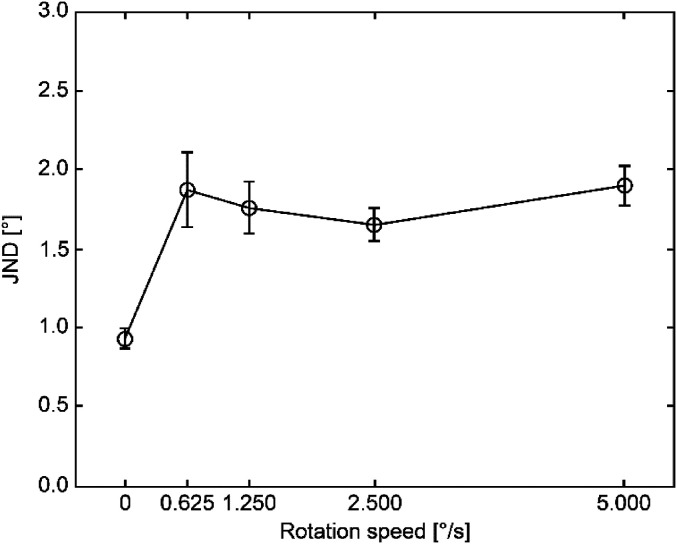
Average JND and standard error values.

While Honda et al. (2020) confirmed the deteriorating effects at low velocities (5°/s), this study confirms the occurrence of these effects at speeds as low as 0.625°/s. More importantly, this study establishes that the PSSA accuracy remains unaffected by the rotational velocity. At the highest rotational speed of 5°/s considered in this study, the participants rotate by 0.15° during the duration of the 30-ms acoustic stimulus. As the rotation speed was constant during each trial, the angle of the sound source changed linearly; therefore, it could act as a source of noise that contributes to the JND. The variance of a continuous uniform distribution is defined as Δ^2^/12, where Δ is the difference between the angles at the start and end of the stimulus, i.e., 0.15. This corresponds to a standard deviation of 0.043°, which is considerably smaller than the JND value of approximately 1.8°. Therefore, the deteriorating effects of sound localization (Cooper et al., 2008; Honda et al., 2016, 2020) are hardly attributable to bottom-up effects, such as ear-input blurring. Honda et al. (2020) reported that the said effect can be attributed to top-down effects such as the participant's conscious perception of motion. The findings of this study suggest the involvement of such a top-down mechanism. During perceptual information processing, the listener should constantly update sound image position information via conscious head/body movement, resulting in an increase in the amount of information to be processed. Taking this into account, information processing becomes difficult due to the higher cognitive load in computing and integrating dynamic changes in the information. Therefore, it can be assumed that a top-down process induced by listeners’ conscious perception of motion may act as a constraint against such information processing overload.

## References

[bibr1-20416695211070616] Cooper J. Carlile S. Alais D. (2008). Distortions of auditory space during rapid head turns. Experimental Brain Research, 191(2), 209–219. 10.1073/pnas.0710837105 18696058

[bibr2-20416695211070616] Honda A. Masumi Y. Suzuki Y. Sakamoto S. (2020). Effect of passive whole-body rotation on sound localization accuracy of listener subjective straight ahead. Acoustical Science and Technology, 41(1), 249–252. 10.1250/ast.41.249

[bibr3-20416695211070616] Honda A. Ohba K. Iwaya Y. Suzuki Y. (2016). Detection of sound image movement during horizontal head rotation. i-Perception, 7(5), 2041669516669614. 10.1177/2041669516669614 27698993 PMC5030746

[bibr4-20416695211070616] Honda A. Shibata H. Gyoba J. Saitou K. Iwaya Y. Suzuki Y. (2007). Transfer effects on sound localization performances from playing a virtual three-dimensional auditory game. Applied Acoustics, 68(8), 885–896. 10.1016/j.apacoust.2006.08.007

[bibr5-20416695211070616] Honda A. Shibata H. Hidaka S. Gyoba J. Iwaya Y. Suzuki Y. (2013). Effects of head movement and proprioceptive feedback in training of sound localization. i-Perception, 4(4), 253–264. 10.1068/i0522 24349686 PMC3859569

[bibr6-20416695211070616] Honda A. Tsunokake S. Suzuki Y. Sakamoto S. (2018). Effects of listener’s whole-body rotation and sound duration on horizontal sound localization accuracy. Acoustical Science and Technology, 39(4), 305–307. 10.1250/ast.39.305

[bibr7-20416695211070616] Iwaya Y. Suzuki Y. Kimura D. (2003). Effects of head movement on front-back error in sound localization. Acoustical Science and Technology, 24(5), 322–324. 10.1250/ast.24.322

[bibr8-20416695211070616] Kawaura J. I. Suzuki Y. Asano F. Sone T. (1989). Sound localization in headphone reproduction by simulating transfer functions from the sound source to the external ear. Journal of the Acoustical Society of Japan, 45(10), 755–766 [in Japanese]. English translation: (1991) *Journal of the Acoustical Society of Japan (e)*, 12(5), 203–216. 10.1250/ast.12.203

[bibr9-20416695211070616] Ogura Y. Sone T. Suzuki Y. (1989). On the errors in estimates when the method of maximum likelihood was applied to the result of psychoacoustical experiment obtained by the constant method. Journal of the Acoustical Society of Japan, 45(6), 441–445 [in Japanese]. 10.1121/1.4733540

[bibr10-20416695211070616] Suzuki Y. Honda A. Iwaya Y. Ohuchi M. Sakamoto S. (2020). Toward cognitive usage of binaural displays. In: Blauert J. Braasch J. (Eds.), The technology of binaural understanding. Modern acoustics and signal processing (pp. 665-695). Springer. 10.1007/978-3-030-00386-9_22.

[bibr11-20416695211070616] Takeshima H. Suzuki Y. Fujii H. Kumagai M. Ashihara K. Fujimori T. Sone T. (2001). Equal-loudness contours measured by the randomized maximum likelihood sequential procedure. Acta Acustica United with Acustica, 87(3), 389–399.

[bibr12-20416695211070616] Thurlow W. Runge P. (1967). Effect of induced head movements on localization of direction of sounds. The Journal of the Acoustical Society of America, 42(2), 480–488. 10.1121/1.1910604 6075941

[bibr13-20416695211070616] Wallach H. (1939). On sound localization. The Journal of the Acoustical Society of America, 10(4), 270–274. 10.1121/1.1915985

